# Pregnancy at 40 years Old and Above: Obstetrical, Fetal, and Neonatal Outcomes. Is Age an Independent Risk Factor for Those Complications?

**DOI:** 10.3389/fmed.2020.00208

**Published:** 2020-05-27

**Authors:** Ana Bouzaglou, Ines Aubenas, Hind Abbou, Stephanie Rouanet, Marie Carbonnel, Paul Pirtea, Jean Marc Bernard Ayoubi

**Affiliations:** ^1^Department of Gynecology and Obstetrics, Hospital Foch, Suresnes, France; ^2^StatEthic SASU, Levallois Perret, France

**Keywords:** advanced maternal age, complication, neonatal, pregnancy, preeclampsia, gestationnal diabetes

## Abstract

**Objectives:** Maternal age has been increasing for several decades with many of these late pregnancies between 40 and 45 years old. The main objective of this study is to assess whether maternal age is an independent factor of obstetric, fetal, and neonatal complications.

**Patients and methods:** A monocentric, French study “exposed-unexposed” was conducted during 11 years in a maternity level IIB. Maternal and perinatal outcomes were studied using univariates and multivariate analysis. We compared women aged 40 and above in a 1:1 ratio with women of 25–35 years old.

**Results:** One thousand nine hundred eighty-two women were 40 or older (mean age: 41.9) on the day of their delivery and compared to other 1,982 women who were aged between 25 and 35 years old (mean age: 30.7) Preeclampsia, gestational diabetes, were significantly higher in the study group (4.6 vs. 1.5% and 14.5 vs. 6.9%, respectively, *p* < 0.001). We found also a significant difference for gestational hypertension (3.1 vs. 1.1% *p* < 0.001), preterm birth (10.4 vs. 6.5% *p* < 0.001), cesarean (16.6 vs. 5.4% for scheduled cesarean, and 50.4 vs. 13.9% for emergency cesarean, *p* < 0.001) and fetal death *in utero* (2.1 vs. 0.5% in the study group, *p* < 0.001). These results were also significantly different in multivariate analysis.

**Conclusion:** A pregnancy after 40 years old is worth considering today as far as the risk factors are controlled and understand by the patient and the obstetrician. However, they have a significantly higher risks of cesarean, preterm delivery, pre-eclampsia, gestational diabetes, and fetal death *in utero* (FDIU). It is therefore the responsibility of the obstetrician to inform correctly these women in a detailed way, to reassure them and to adapt the monitoring of their pregnancy accordingly.

## Introduction

Late pregnancies have been a sensitive subject in society and in the medical field since a couple years. Indeed, maternal age has been increasing for several decades with many of these late pregnancies between 40 and 45 years old (1). In France, the latest INSEE report shows that the proportion of pregnant women over 35 years rose from 19.3 to 21.3% between 2010 and 2016. This report states that about 5% of women who give birth are 40 years old or older. The age of first pregnancy increased from 29.5 in 2003 to 30.4 in 2016 (2–4). Decades earlier, a pregnancy was considered “late” if it was obtained after 35 years, today the threshold has shifted to 40 years or even 43 or 45 years according to the scientific literature (3, 5–7). This is explained by a societal evolution marked by a constantly increasing level of studies by women who have more responsibilities at work and therefore delay their project of childbearing giving their first priority to their professional career (1). In addition, the increasing development of medically assisted procreation (ART), particularly with access to oocyte donation in European countries, has recently been re-established (6, 8, 9).

According to many studies, advanced maternal age is often associated with several obstetrical complications (gestational diabetes, hypertension, pre-eclampsia (1–3, 5, 6, 10), and fetal complications (growth retardation, prematurity, fetal malformation) (1–3, 5, 6, 10).

However, the ratio of maternal mortality (RMM) remains stable at 10.3/100,000 births, according to the 5th report of the national perinatal survey on maternal deaths in France.

The age of women is an uncontested factor of maternal death risk (11). Between 2010 and 2012, nearly 30% of maternal deaths occurred in women aged 35–39 years old (for 17% of live births in this age group), vs. 10% for women aged 40 and over (for 5% of live births in this age group) (2). Today, maternal complications are managed more effectively than some years ago. Maternal mortality in France is 80 women per year. Given the small numbers, these evolutions should be interpreted with caution.

Thus, many studies suggest that maternal age increases the risk of these complications, but sometimes not statistically significantly because of small samples (3).

Moreover, there are few studies taking into account the very advanced maternal age considered in the literature around 43 and 45 years old (4, 9, 12, 13).

Studies are still few and obstetric and pre-conceptional monitoring of these patients is not yet standardized.

Therefore, we decided to carry out an additional study with a sample of women over 40 years old to confirm this trend.

The main objective of this study is to assess whether maternal age is an independent factor of obstetric, fetal, and neonatal complications.

## Patients And METHODS

### Design of the Study and Participants

This is a French cohort “exposed – unexposed,” monocentric study performed in a maternity level IIB (hospital Foch, Suresnes). All women aged 40 or older who gave birth or who had a late miscarriage or medical termination between January 1, 2006 and December 31, 2017 were extracted from the DIAMM database and included in the exposed group. The unexposed group was constituted as follows: each woman aged 40 or over was matched with a woman aged 25–35, for whom delivery or late miscarriage or medical termination of pregnancy was performed consecutively (delivery number).

The characteristics of each pregnancy were extracted from the DIAMM software: maternal characteristics: medical history (High Blood Pressure—Type I or II diabetes—thromboembolic disease (defined as any history of venous or arterial thrombosis and/or the presence of anti-phospholipid syndrome), parity, body mass index -BMI-, smoking, geographical origin, assisted reproduction technology (ART) (artificial insemination, IVF, ICSI, gamete donation), type of pregnancy (single or twin), the method of delivery, and indications for cesarean section (CS).

Adjustment factors for preeclampsia are BMI, presence of at least one medical history, mode of conception and type of pregnancy. For gestational diabetes, the adjustment was made on BMI, the mode of conception and the type of pregnancy. For pregnancy HTA, the adjustment was made on BMI and type of pregnancy. For prematurity the adjustment factors were parity, ethnic group, type of pregnancy, pre-eclampsia, and gestational hypertension. Finally, for the variable “cesarean section” the adjustment was made for BMI, geographic origin, conception mode, and type of pregnancy.

### Objectives

The main objective of the study is to determine the incidence of obstetric, fetal, and neonatal complication and to assess whether age is an independent factor of these complications.

The secondary objectives are to determine whether there is an association between some complications (pre-eclampsia, gestational diabetes, prematurity) and the conception mode associated with the type of pregnancy (singleton or twin).

The obstetrical complications studied are gestational hypertension (defined as systolic >140 mmH and/or diastolic >90 mmHg without proteinuria), pre-eclampsia (systolic >140 mmHand/or diastolic >90 mmHg associated with a proteinuria of 24 h >300 mg), gestational diabetes (defined according to the recommendations of the 2015 CNGOF), cesarean section (CS), admission of women to the intensive care unit during their pregnancies, postpartum hemorrhage (loss of more than 500 cc of blood within 24 h after vaginal delivery or CS) and blood transfusion.

The fetal complications studied are intrauterine growth retardation (IUGR) (defined as having an estimation of fetal weight <5e p) and fetal death *in utero* (FDIU).

The neonatal complications studied were prematurity (birth before 37 weeks), pH at birth (acidosis with pH <7.10), APGAR score (<7), and pediatric care just after the birth.

## Statistics

Continuous variables are reported as mean ± standard deviation (sd). Categorical variables are reported as number and percentage (percentages were calculated excluding missing data) and are compared by Chi-2 test or Fisher's exact test, as appropriate. Missing data were not treated.

Incidence of each complication (obstetric, fetal, or neonatal) was presented with its associated 95% confidence interval (Wilson method). The association between age and obstetric, fetal, or neonatal complications was assessed using univariate analysis. When age was significantly associated with a complication (*p* < 0.05), a multivariate analysis was performed adjusted for mother and/or pregnancy characteristics significantly associated with this complication in univariate analysis (*p* < 0.2). Univariate characteristics tested were, BMI, geographical, smoking, parity, presence of at least one medical history (hypertension, diabetes, venous thromboembolic disease/vascular pathology/lupus), mode of conception, type of pregnancy, delivery period (2006–2009 vs. 2010–2017) and for prematurity, gestational diabetes, pregnancy-induced hypertension, and preeclampsia. Multivariate analyses were performed using a logistic regression model (stepwise selection). The results are interpreted in terms of adjusted odd ratios with their associated 95% confidence interval.

A *p* < 0.05 was considered significant unless otherwise specified. All statistical analyses were performed with SAS release 9.4 (SAS Institute Inc, Cary, NC) statistical software package.

## Results

**Figure 1:**
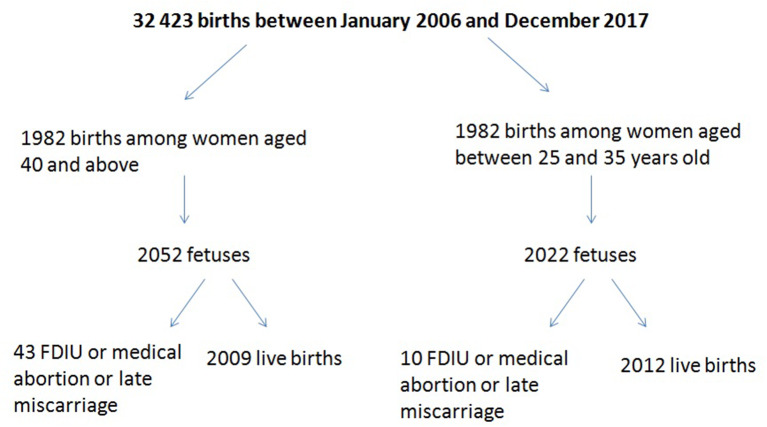
Flow chart.

Thirty-two thousand two hundred forty-three patients gave birth between January 2006 and December 2017, of whom 1,982 were 40 or older on the day of delivery (Figure 1). The rate of late pregnancies in our study doubled from 2006 to 2016.

The demographic and obstetric characteristics for the study and the control groups are given in Table 1. The analysis of those characteristics did not find a significant difference between the two groups concerning: ethnic group, medical history, or smoking. There was a significant difference between the two groups for the access to ART, Body mass index (BMI), and type of pregnancy (*p* < 0.001) (Table 1).

**Table 1:** The demographic and obstetric characteristics for the study and the control groups.

	**40 years and over** **(*N* = 1,982)**	**25–35 years (*N* = 1,982)**	***p***
Maternal age (standard deviation)	41.9 (1.8)	30.7 (2.6)	
Ethnic group			0.115
Europe	1,272 (65.7%)	1,274 (66.5%)	
Africa	530 (27.4%)	478 (24.9%)	
Overseas departments and territories	35 (1.8%)	46 (2.4%)	
Others	98 (5.1%)	118 (6.2%)	
BMI (kg/m2)			<0.001
- <18	51 (2.6%)	59 (3.0%)	
- [18;25]	1,336 (67.8%)	1,483 (75.0%)	
- [25;30]	398 (20.2%)	319 (16.1%)	
- [30; 35]	143 (7.3%)	87 (4.4%)	
- [35; 40]	31(1.6%)	25 (1.3%)	
- ≥40	11 (0.6%)	4 (0.2%)	
Smokers	158 (8.0%)	184 (9.3%)	0.141
Parity - primiparity	730 (36.9%)	1,444 (72.9%)	<0.001
Multifetal gestation			<0.001
- single	1,601 (81.8%)	1,868 (94.3%)	
- twins	356 (18.2%)	112 (5.7%)	
Mode of conception			
- Spontaneous pregnancy	1,601 (81.8%)	1,868 (94.3%)	
- Pregnancy with ART	356 (18.2%)	112 (5.7%)	<0.001
History of high blood pressure	107 (5.4%)	92 (4.6%)	0.272
History of diabetes	62 (3.1%)	63 (3.2%)	0.932
History of thrombo-embolic event, vascular pathology, MTEV, Lupus	43 (2.2 %)	47 (2.4%)	0.673
At least one risk factors^*^	195 (9.9%)	179 (9.0%)	0.379

* *Any patient with hypertension and/or diabetes and/or Thromboembolic event/vascular pathology/lupus*.

Table 2 compares obstetric, fetal, and neonatal complications in univariate analysis. There is a significantly higher rate of obstetric pathology with 4.6% of pre-eclampsia for women aged 40 and over compared with 1.5% in the control group and 3.1 vs. 1.1% for gestational hypertension. There is also a significant difference for transfusion. With regard to gestational diabetes, there was 14.5% of women aged 40 and over, compared to 6.9%. However, no significant difference was found for postpartum hemorrhage and transfer to an intensive care unit. During the 11 years studied, no maternal deaths were observed.

**Table 2:** Obstetrical, fetal, and neonatal outcome.

	**40 years and over** **(*N* = 1,982)**	**25–35 years** **(*N* = 1,982)**	p^***^
Preeclampsia	90 (4.6 %)	30 (1.5%)	<0.001
	[3.7%; 5.6%]	[1.1%; 2.2%]	
Gestational hypertension	61 (3.1%)	21 (1.1%)	<0.001
	[2.4%; 3.9%]	[0.7%; 1.6%]	
Gestational diabetes	286 (14.5%)	136 (6.9%)	<0.001
	[13.0%; 16.1%]	[5.8%; 8.1%]	
Post-partum hemorrhage	81 (4.1%)	63 (3.2%)	0.121
	[3.3%; 5.1%]	[2.5%; 4.0%]	
Blood transfusion	17 (0.9%)	6 (0,3%)	0.021
	[0.5%; 1.4%]	[0.1%; 0.7%]	
Admission of the mother in intensive care unit	11 (0.6%)	6 (0.3%)	0.224
	[0.3%; 1.0%]	[0.1%; 0.7%]	
Delivery mode			<0.001
- Scheduled cesarean	328 (16.6%)	108 (5.4%)	
	[15.0%; 18.3%]	[4.5%; 6.5%]	
- Emergency cesarean	404 (20.4%)	275 (13.9%)	
	[18.7%; 22.2%]	[12.4%; 15.5%]	
- Vaginal delivery	1249 (63.0%)	1599 (80.7%)	
Prematurity (<37 wk)	206 (10.4%)	128 (6.5%)	<0.001
	[9.1%; 11.8%]	[5.5%; 7.6%]	
IUGR or fetal malformation ¤	167 (8.3%)	133 (6.6%)	0.039
	[7.2%; 9.6%]	[5.6%; 7.8%]	
Apgar score less or equal to <7¤	43 (2.1%)	38 (1.9%)	0.573
	[1.6%; 2.9%]	[1.4%; 2.6%]	
Neonatal pH in the umbilical cord <7,10¤	82 (4.3%)	90 (4.6%)	0.591
	[3.5%; 5.3%]	[3.8%; 5.7%]	
New-born intensive care¤	234 (11.7%)	197 (9.8%)	0.054
	[10.3%; 13.2%]	[8.6%; 11.2%]	
Fetal death in utero^*^	43 (2.1%)	10 (0.5%)	<0.001
	[1.6%; 2.8%]	[0.3%; 0.9%]	

* in 2052 fetuses for over 40s and 2022 fetuses in 25–35 years ¤on 2009 fetuses for over 40s and on 2012 fetuses on 25–35s

*** *Chi-2 test*.

For fetal and neonatal outcomes, there is a significantly higher proportion of FDIU, IUGR, and prematurity. There are comparable rates between the two groups for Apgar score, cord pH, and pediatric care just after birth.

Maternal and fetal complications in multivariate analysis are given in Table 3.

**Table 3:** Association between age and complications (uni and multivariate analysis).

	**Univariate** **OR [IC 95%] (*p*-value)**	Multivariate^**^ **OR ajusté [IC 95%] (*p*-value)**
Preeclampsia	3.10 [2.04; 4.71] (<0.0001)	2.46 [1.58; 3.81] (<0.0001)
Gestational diabetes	2.30 [1.85; 2.85] (<0.0001)	2.49 [1.61; 3.85] (<0.0001)
Gestational hypertension	2.98 [1.81; 4.91] (<0.0001)	2.59 [1.57; 4.30] (0.0002)
Preterm birth <37SA	1.68 [1.34; 2.11] (<0.0001)	1.55 [1.19; 2.02] (0.0010)
Cesarean	2.35 [2.03; 2.71] (<0.0001)	2.07 [1.78; 2.42] (<0.0001)
Fetal death *in utero*	4.31 [2.16; 8.60] (<0,0001)	4,59 [2.20; 9.55] (<0,0001)

Selection of variables p ≤ 0.15

** *Stepwise model (input threshold 0.15, output threshold 0.05) Note: the multivariate model was performed on 3,821 pregnancies (143 missing data)*.

Among patients of 40 years old and above, obstetric complications are significantly more frequent, with an increased risk of gestational diabetes (OR = 2.49 (95% CI 1.61, 3.85) < 0.0001), pre-eclampsia (2.46 [1.58; 3.81] < 0.0001), gestational hypertension (2.59 [1.57, 4.30] 0.0002) and cesarean delivery (2.07 [1.78, 2.42] < 0.0001) (Table 3). Fetal risk of FDIU was significantly greater in patients 40 years of age or older (OR = 4.31 95% CI [2.16, 8.60] with *p* < 0.0001). Similarly, for prematurity, where the difference observed is significant between the two groups (*p* = 0.0010).

The secondary objectives are specified in Table 4. There is a significant difference for preeclampsia and prematurity. No significant difference was observed for gestational diabetes. We find a rate of pre-eclampsia in patients using ART higher than in patients having a spontaneous pregnancy among the women of 40 and above (for multifetal gestation and ART 22.5 vs. 16.7% and single pregnancy with ART 6.6 vs. 3.3%). We did not find a significant increase in gestational diabetes regardless of the type of conception (ART vs. spontaneous pregnancy).

**Table 4:** Description of obstetric complications in patients aged 40 and above, type of pregnancy and use of PMA.

	**Multifetal gestation and ART (*N* = 40)**	**Single pregnancy and ART (*N* = 316)**	**Multifetal gestation without ART (*N* = 30)**	**Single pregnancy without ART (*N* = 1,571)**	***p***
Preeclampsia	9 (22.5%)	21 (6.6%)	5 (16.7%)	52 (3.3%)	<0.001 (F)
OR [95% IC]^*^	8.48 [3.84; 18.72]	2.08 [1.23; 3.50]	5.84 [2.15; 15.87]	1	
Gestational diabetes	7 (17.5%)	52 (16.5%)	7 (23.3%)	217 (13.8%)	0.242 (F)
OR [95%IC]^**^	1.32 [0.58; 3.03]	1.23 [0.88; 1.71]	1.90 [0.80; 4.47]	1	
Preterm <37wk	22 (55.0%)	38 (12.0%)	14 (46.7%)	126 (8.0%)	<0.001 (F)
OR [95%IC]^***^	14.02 [7.33; 26.82]	1.57 [1.07; 2.30]	10.04 [4.79; 21.03]	1	

* *Association Pre-eclampsia and Type of Pregnancy and ART in women of 40 years and older*.

** *Association Gestational Diabetes and Pregnancy Type and ART in Women of 40 Years and above*.

*** *Association Gestational Age < 37 Weeks and Type of Pregnancy and ART in Women Age 40 and above*.

## Discussion

Our study shows that advanced maternal age is an independent risk factor for obstetric and neonatal complications (14, 15). In fact, multivariate analysis found significant results for three of the most common pregnancy-related diseases: gestational hypertension, pre-eclampsia, and gestational diabetes. Our large sample significantly confirms the occurrence of pre-eclampsia in women aged 40 and above, unlike some studies with small samples that did not find this result in multivariate analysis (3).

Moreover, there is a higher risk of pre-eclampsia when the patient has some other risk factor such as twin pregnancy or medical history (hypertension and/or diabetes and/or VTE/vascular disease/lupus) (16, 17). Even more, these women with advanced maternal age are at higher risk of developing cardiovascular and nephrological diseases in the long term (18). In the case of tobacco, it has not been found as an independent risk factor, which can probably be explained by a significant underestimation of women reporting smoking during pregnancy.

The high proportion of cesareans in the study group of women over 40 is due to some contributing factors. On one hand, the percentage of scheduled cesareans is higher because there is a higher prevalence of uni or multi-cicatricial uterus.

Cesareans for high maternal age or for maternal request finally represented a small sample (22 women/1,982, 1% in the exposed group vs. 1/1,982 in the unexposed group). There was also a higher rate about the emergency cesareans deliveries in the study group over 40 years old. Several physiological hypotheses have been mentioned in previous studies (2, 3): a higher rate of dystocia presentation and scarred uterus, uterine contractility less effective than for a woman aged 25–35. In our sample, the most common indications for CS were abnormalities of cardio-fetal rhythm and cervical dystocia (19). It is likely that CFR abnormalities are more severely judged by the obstetrician, in the context of older patients, especially if the pregnancy is a result of ART, putting some women at a risk of cesarean that is not always justified (20). In total, this large proportion of CS in women 40 years and older has also been shown in other studies (3, 4, 17, 20–22). However, these results should be taken with caution because some indications for CS are inherent to the protocols practiced in our unit.

The association between advanced maternal age and fetal deaths *in utero* should also be taken into account. Among those 43 FDIUs, we have looked at every medical files of those women and we did not find any events that could explained this high number. Indeed, among the 43 FDIUs in women aged 40 and above, there were no more patients using ART, nor more patients with obstetric pathology. This can be explained by a small number of FDIU. The only common point in our study group was the advanced maternal age. In these circumstances, instead of worrying the patients, it might be more appropriate to give them clear and reassuring information while performing a pre-conception close monitoring and throughout the pregnancy. This would help detect and manage these complications much earlier. In addition, with the advanced technology, several risks are now monitored using non-invasive prenatal screening or even the pre-implantation diagnosis (23–26).

The incidence of maternal complications is likely to increase over time due to increased maternal age. It will be difficult to reduce the incidence of these complications, but we can reduce the serious complications of preeclampsia, gestational diabetes (such as eclampsia, and macrosomia) through appropriate management (induce delivery before 41 weeks, close monitoring of the fetus) (27, 28).

With regard to neonatal complications, few significant differences were found in our study, as well as in the literature (29, 30). This is partly explained by the fact that several obstetrical factors can interfere without being related to age (the length of the delivery, abnormalities of the RCF, chorioamnionitis (3, 31, 32).

Our study has several advantages. On the one hand, our study was done on a large sample, with data processing from medical records with a complete search for missing data. International and European studies with large samples use public health registers, thereby providing a lot of information on the characteristics of the population (5, 10). However, this is often at the expense of information such as the type of delivery, the methods of neonatology care which are sometimes different in hospitals.

On the other hand, we took a period of 11 years, to check if there had been a difference in daily practices. We did not notice any difference between the periods 2006–2010 and 2011–2017 except for the increasing number of patients who have access to ART.

On the other hand, we matched each patient aged 40 and above to a patient aged between 25 and 35 whose delivery number followed the patient case. Indeed, this allowed us to limit as far as possible all the variability of practices on the delivery route (natural delivery vs. cesarean, neonatal care). We also had the advantage of separating fetuses, newborns, and mothers, which has not been realized in other studies, and which may lead to a classification bias regarding perinatal outcomes.

Our study is yet limited by its monocentric character and retrospective aspect. In addition, Foch Hospital has an ART center, so our sample probably contained more patients using these techniques. However, we had the opportunity to have 18.2% of women over 40 using ART. This allowed us to highlight the significant increase in preeclampsia and prematurity in patients over 40 years of age who have used ART. After 44 years, 1 out of 2 women used the ART. This rate is surely underestimated because there is a large number of patients who voluntarily omit to declare their use of ART in particular the use of donated oocytes (33).

It is especially remembered that maternal complications occurring decades ago are less morbid today than before (22, 34). Screening and management of maternal and neonatal complications are progressively improving, and a high-risk pregnancy at age 40 in the 1980s should no longer discourage patients and obstetricians in 2020.

## Conclusion

The desire to become pregnant after 40, 45, or even 50 years will most likely continue to develop in our society. Thus, it is important to multiply the studies on this subject in order to inform these patients as much as possible. Our study confirms the current trend among this group of women over 40 years of age to know that advanced maternal age leads to more significant obstetric complications.

However, in the absence of cumulative risk factors and with appropriate management, a pregnancy after 40 years can go well-physiologically without having a higher maternal mortality or neonatal morbidity.

It is therefore the duty of the obstetrician to inform these women in an enlightened way, to reassure them and to adapt the monitoring of their pregnancy according to the risk factors, the modality of conception, and the multifetal gestation.

## Data Availability Statement

All datasets generated for this study are included in the article/supplementary material.

## Author Contributions

AB and IA created and organized the database and wrote several sections of the manuscript. JA, HA, MC, and PP contributed conception and design of the study. SR performed the statistical analysis and wrote the statistics section of the manuscript. All authors contributed to manuscript revision, read and approved the submitted version.

## Conflict of Interest

Statistics calculation were performed by StatEthic SASU. JA was employed by company StatEthic SASU. The remaining authors declare that the research was conducted in the absence of the commercial relationships that could be construed as a potential conflict of interest.
